# Changes in voiding behavior in a mouse model of Alzheimer’s disease

**DOI:** 10.3389/fnagi.2015.00160

**Published:** 2015-08-25

**Authors:** B. T. Biallosterski, J. Prickaerts, M. S. Rahnama’i, S. de Wachter, G. A. van Koeveringe, C. Meriaux

**Affiliations:** ^1^Department of Urology, University Hospital MaastrichtMaastricht, Netherlands; ^2^Department of Neuro-Urology, School for Mental Health and Neuroscience, Maastricht UniversityMaastricht, Netherlands; ^3^Department of Psychiatry and Neuropsychology, School for Mental Health and Neuroscience, Maastricht UniversityMaastricht, Netherlands; ^4^Department of Urology, University Hospital AntwerpenEdegem, Belgium

**Keywords:** Alzheimer’s disease, mouse model, voiding behavior, anxiety, locomotor behavior

## Abstract

Besides cognitive decline and behavioral alteration, urinary incontinence often occurs in patients suffering from Alzheimer’s disease (AD). To determine whether the transgenic mouse model of AD, APP/PS1 (APP^SL^/PS1^M146L^) mouse, shows alteration of the urinary bladder function and anxiety, as for patients with AD, we examined the urinary marking behavior in relation to affective behavior. At 18 months of age voiding behavior of APP/PS1 and wild type (WT) mice was assessed by using a modified filter paper assay in combination with video tracing, with the cage divided into a center and corner zones. Anxiety-related behavior and locomotion were respectively tested in an elevated zero maze (EZM) and an open field (OF). The APP/PS1 mice urinated more in the center zone than the WT mice. The total volume of markings was significantly lower in the APP/PS1 mice. In both groups, the average volume of a marking in the corner zone was larger than in the center zone. In the EZM, the APP/PS1 mice spent less time in the open arms of the arena, considered as anxiogenic zones, than the WT mice. During the OF task, the APP/PS1 mice covered a longer distance than the WT mice. These findings show that the APP/PS1 mice have a different voiding behavior compared to the WT mice, i.e., urinating with small volumes and voiding in the center of the cage, and suggest that increased locomotor activity and anxiety-related behaviors are factors in the change in voiding pattern in the APP/PS1 mouse.

## Introduction

In neurodegenerative disorders, like dementia and Alzheimer’s disease (AD), patients can suffer from impairment of bladder function (Ransmayr et al., 2008). Incontinence often emerges when dementia has developed into a moderate disease stage (Han and Wang, 2008). In general, people with sufficient cortical function commence voiding at an acceptable moment after going to a suitable location with the appropriate measures taken i.e., in a toilet and partially undressed. However, patients with dementia and AD especially can demonstrate uninhibited and unembarrassed voiding at an inappropriate place and/or time. This behavior is often referred to as functional incontinence, and could be caused by dysfunctions at several levels of control. The general belief is that this incontinence is not derived from an abnormality in the lower urinary tract or its innervation, but from deficiencies in cholinergic neurotransmission in the cortical and subcortical areas of the central nervous system which then consequently leads to problems in locomotion and cognitive decline (Jirovec and Wells, 1990). Cognitive severity and decline in dementia have been shown to significantly correlate to behavioral alterations, such as anxiety (Serra et al., 2010). Several studies have linked anxiety to voiding dysfunction (Fan et al., 2008; Serra et al., 2010). Many patients with AD also show signs of detrusor overactivity upon cystometric evaluation (Sugiyama et al., 1994; Ransmayr et al., 2008). In the latter condition, dysfunctions in the integrated (neuro)-physiology of the bladder itself may contribute to this disabling condition. This is supported by a previous report from our group in which structural changes have been found in the bladder of a transgenic mouse model of AD (Biallosterski et al., 2010). Consequently, all these factors could be responsible for the altered voiding behavior seen in patients with AD. To gain insight into bladder function rodents are often studied using metabolic cages, allowing voiding frequency and volume to be measured while urine can be collected and analyzed (Wood et al., 2001; Stechman et al., 2010). However, this method does not provide any information on the voiding behavior of freely moving animals. Because AD is correlated with both anxiety and incontinence (Ransmayr et al., 2008), we wanted to investigate both the affective and the voiding behavior of transgenic APP^SL^/PS1^M146L^ (APP/PS1) mice, a mouse model of AD. We have previously used these mice, which have well-established cognitive impairment, clearly recognizable plaques and tangles and structural bladder changes (Biallosterski et al., 2010; Vanmierlo et al., 2011). We hypothesize that this mouse model of AD displays an altered voiding behavior compared to wild type (WT) mice. Voiding behavior of APP/PS1 and WT is compared by using a modified filter paper assay in combination with video-assisted tracing in time of moving and voiding patterns.

## Materials and Methods

Animals were housed individually within a temperature-controlled environment with reversed 12-h light/12-h dark cycle (lights off from 7.00 h) and standard chow and water available ad libitum at all times. All animals were subjected to one experiment per day for three consecutive days. The experiments were performed in the following order: (1) “Voiding behavior task” (VBT); (2) “Elevated zero maze task” (EZM); and (3) “Open field task” (OF), respectively. This study was approved by the Animal Ethics Board of Maastricht University, Netherlands.

### Animals

Mice with gene-targeted expression of the APP mutant encoding the Swedish/London-FAD mutations were generated as described previously (Kohler et al., 2005) and crossbred with PS1^M146L^ transgenic mice to generate APP^SL^/PS1^M146L^ mice (APP/PS1). One-year-old WT C57BL/6NCrl mice were purchased from Charles River Laboratories (L’Arbresle, France) and allowed to age in the same animal facility. At 18 months of age animals were divided into two groups, APP/PS1 (*n* = 7; 5 females, 2 males) and WT (*n* = 10, males) mice were taken into experiment.

### Voiding Behavior Task

**Figure 1:**
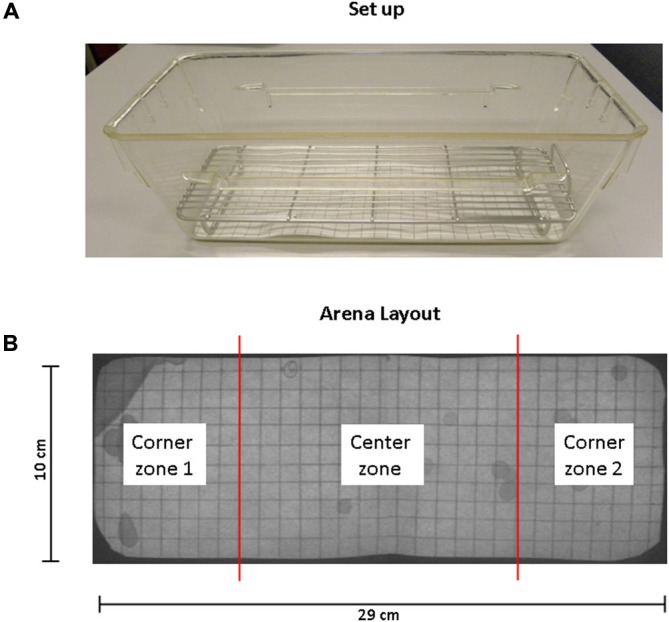
**Standard set up and arena layout for voiding behavior task (VBT). (A)** This photograph shows the cage, grid and filter paper as used in the VBT. The mouse is placed in the center of the cage at the start of the experiment. A metal cage top closes the cage and provides for food and water. **(B)** Arena layout. UV photograph showing filter paper on the bottom of the conventional mouse cage. The floor of the cage measures 29 cm by 10 cm; grid spacing is 1 cm^2^ in both directions. The total area of corner zones 1 and 2 combined equal the area of the center zone.

**Figure 2:**
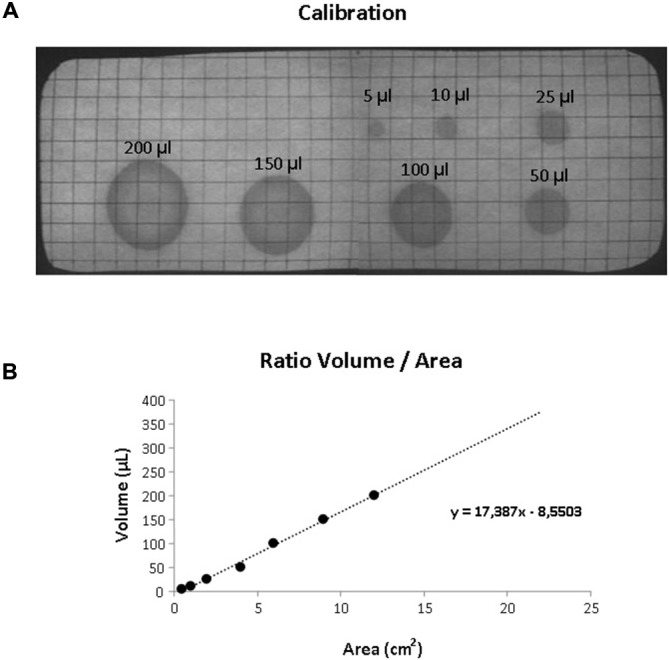
**Volumetric calibration. (A)** UV photograph of the filter paper showing standardized volumes of urine with the corresponding area. **(B)** Scatter plot illustrating the relationship between volume and surface area of the urinary markings. The dotted line illustrated the linear trend (equation shown in the graph).

Mice were habituated to the test cage for approximately 5 h, 24 h prior to testing to minimize the increased urinary marking when mice are placed in a novel environment (Maruniak et al., 1974). Animals were placed individually in standard cages for five hours (332 × 150 × 130 mm—floor area 290 cm^2^) with the floor lined with filter paper (Bench coat paper, VWR international, Amsterdam, Netherlands). Animals were raised of the floor by 30 mm to avoid direct contact with the filter paper by placing a standardized metal walking grid in the cage (grid 1144B-150, Techniplast, Buguggiate, Italy; Figure 1A). Urinary markings on the paper were allowed to dry for 12 h, after which they were illuminated with ultraviolet light, and photographed (Desjardins et al., 1973). The experiment was recorded on video with a low level light from below illuminating the filter paper. For analysis of behavior the cage was divided into three zones, the two corners were analyzed together as* corner zone* and the third zone, the center as the *center zone*. The surface area of these zones was equal (Figure 1B). The frequency and pattern of urinary markings were quantified within each zone, the volume for each marking calculated and the total distance moved and overall time spend in each zone recorded. In addition, the time point of each marking was recorded allowing easy identification and discrimination of different overlapping markings. In analogy with Hu et al. (2000) a linear relationship was found between spot area and the volume of the urinary marking, resulting in a ratio of 8.84 μl/cm^2^ (Figure 2). The physiochemical composition and water proportion of urine might contribute to the variance of the spot size. However, no difference is expected due to general variance in water and solid substances ratio in urine since no significant difference in spots size was observed throughout the different trials of voiding behaviour task during which water was not provided to mice.

### Elevated Zero Maze Task

The EZM was performed in order to study anxiety-related behavior (Shepherd et al., 1994). The test was carried out on a maze constructed of black plastic, transparent for infrared light. The circular runway was 50 cm in diameter, with a pathway width of 5 cm placed 20 cm above floor level. The maze was equally divided in two opposite open and two opposite closed parts enclosed by 50 cm high sidewalls. To prevent falls, a 5 mm high rim lined the open parts. A mouse was placed on one open arm, facing one of the closed arms of the maze and was allowed to explore the arena for 5 min. At the end of each experiment the surface was cleaned with 70% ethanol. Mice were tracked under low light conditions with an infrared video tracking system (Ethovision, Noldus, Netherlands) to determine the total distance moved and time spent in zones (closed or open). The partitioning of time between open and closed regions was the principal index of anxiety/fearfulness.

### Open Field Task

The OF was conducted to analyze spontaneous locomotor behavior. The assessment was conducted in a square, Plexiglas box (25 cm × 25 cm × 30 cm), with an open top and gray floor. The floor of the arena was divided into center, periphery, and corner zones, defined by lines spaced 5 cm from the sidewalls. A mouse was placed in the center of the arena and allowed to move freely. The movements and position of the animals were recorded and registered automatically by a computerized system (EthoVision, Noldus, Netherlands) in order to determine the time spent in the different zones and the total distance moved during the 10 min trial. After each experiment the surface was cleaned with 70% ethanol.

### Statistics

All data are represented as mean and standard error of mean (SEM). All statistic analyses were done using the Statistical Package for the Social Sciences (SPSS 15.0 software, Chicago, IL, USA). Chi-square analysis was conducted for the VBT to compare the location of urinary markings within the groups. Between the groups, urinary markings, inter-marking interval were compared using a non-parametric Mann–Whitney *U* test. The accepted level of statistical significance was determined on *p* < 0.05 for all analyses. The EZM and OF were analyzed over time using the unpaired 2-sided Student *t*-test with location and distance moved as variables. One animal from the WT group was excluded from further analysis because of extreme anxiety-related behavior in the EZM (continuous freezing).

## Results

### Voiding Behavior Task

There was no difference found in body weight between the groups (WT vs. APP/PS1; 25.8 ± 1.3 vs. 24.2 ± 1.1 g, *p* = 0.261). Considering the limited availability of animals at the time of the study, both male and female mice were included in this study. However, no difference was observed within the different studied parameters between the genders.

**Figure 3:**
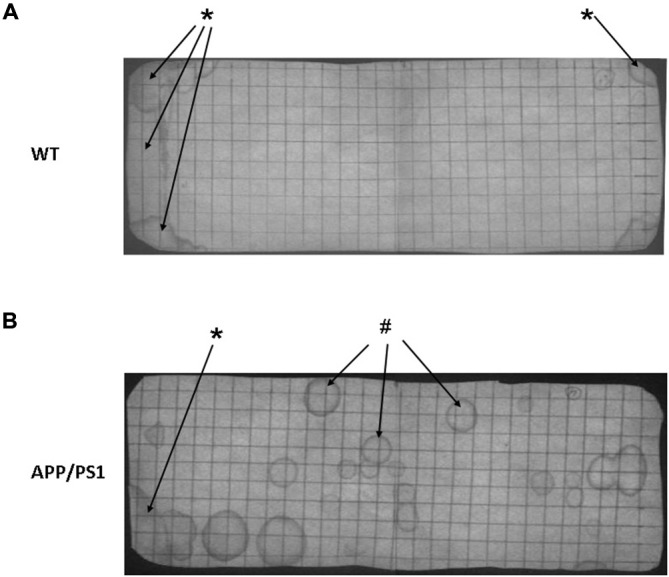
**Representative photographs of a voiding behavior task (VBT) filter paper of a wild type (WT) mouse (A) and an APP/PS1 mouse (B). (A)** The VBT of the WT mouse has a distinct pattern with a high marking density in both corner zones (*arrows), whereas no urinary markings are visible in the center zone. **(B)** In the APP/PS1 mouse urinary markings occur all across the floor area, in both corner zones but also in the center zone (^#^arrows). Similar to the WT mouse non-circular urinary markings were observed in the corner zones (*arrow). These results were representative for all other mice in both APP/PS1 and WT groups.

Figure 3 shows photographs of filter paper of the VBT of APP/PS1 mouse (Figure 3B) or WT mouse (Figure 3A). Under UV light the urinary markings were clearly visible as dark halos on a light background. In general, two types of urinary marking behavior could be identified from the video tracking analysis. Common were non-circular markings seen in the corner zone, whereby the mouse (both WT and APP/PS1) pushed its backside against a wall of the cage and urinated (Figure 3, *arrows). In contrast, circular markings occurred away from the walls and could be found anywhere in the cage. Video tracking analysis showed that they appeared when the mice made a short pause in their locomotion (Figure 3, ^#^arrows).

**Figure 4:**
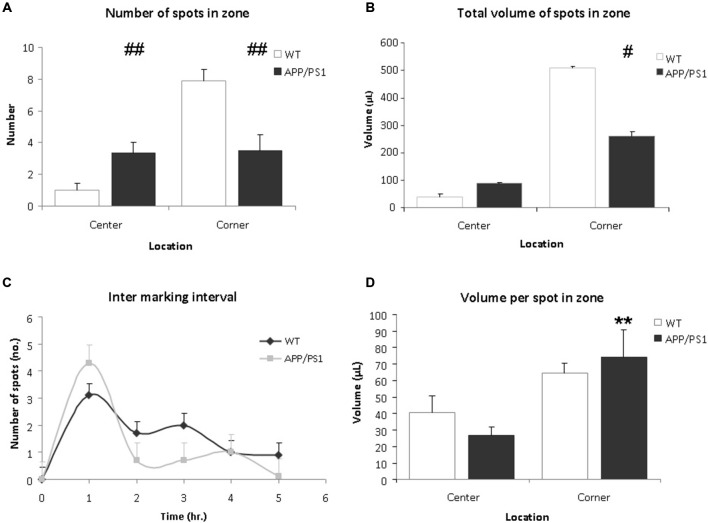
**Voiding behavior task (VBT); Voiding parameters. (A)** The average number of markings in a zone. In corner zone, the number of urinary markings was significantly higher for the WT mice than the APP/PS1 mice. The APP/PS1 mice significantly urinated more in the center zone compared to the WT mice. Values are represented as means ± SEM for the number of animals analyzed (WT *n* = 9 and APP/PS1 *n* = 7; different from WT ^##^*p* < 0.01). **(B)** The total volume of urinary markings in a zone. Compared to APP/PS1 mice, the volume of urinary markings for WT mice were larger in the corner zone but not in the center zone. Values are represented as means ± SEM for the number of animals analyzed (WT *n* = 9 and APP/PS1 *n* = 7; different from WT ^#^*p* < 0.05). **(C)** The inter-marking interval shows the average number of markings per hour. No difference was observed between the groups, the frequency of micturition was relatively high initially and decreased gradually during the trial. Values are presented as means ± SEM. **(D)** The average volume of a spot in a zone. In both center and corner zones, there was no significant difference in average volume of urinary markings between the WT and APP/PS1 mice. In APP/PS1 group, the average volume of a marking in the corner zone was larger than in the center zone. Values are represented as means ± SEM (APP/PS1 group, corner vs. center, **p < 0.01).

Upon analysis the VBT of the APP/PS1 mice appeared significantly different from WT mice. Firstly, the number of markings in the center zone of the APP/PS1 mice was significantly higher compared to the WT mice (Figure 4A, WT vs. APP/PS1; 1.0 ± 0.4 vs. 3.3 ± 0.7, *p* = 0.009). Conversely, APP/PS1 mice urinated less frequently in the corner zone compared to WT mice (Figure 4A, WT vs. APP/PS1; 7.9 ± 0.7 vs. 3.5 ± 1.0, *p* = 0.003). No difference was found between the individual mice of the APP/PS1 group.

Secondly, the total volume of markings for the APP/PS1 mice was lower than for the WT mice (WT vs. APP/PS1; 548.9 ± 5.7 μL vs. 348.7 ± 9.4 μL, *p* = 0.036). In corner zone, the total volume of markings for WT mice was significantly larger than for APP/PS1 mice whereas in center zone, the opposite was observed (Figure 4B, corner WT vs. APP/PS1; 508.7 ± 6.2 μL vs. 259.9 ± 16.4 μL, *p* = 0.014 and center; WT vs. APP/PS1; 40.2 ± 10.4 μL vs. 88.9 ± 5.09 μL, *p* = 0.28).

Concerning the average volume of a marking, in the WT group, no significant difference was observed between the corner zone and the center zone (Figure 4D, corner vs. center; 64.4 ± 6.2 μL vs. 40.2 ± 10.4 μL, *p* = 0.18). Interestingly, in the APP/PS1 group, the average volume of a marking in the corner zone was greater than in the center zone (Figure 4D, corner vs. center; 74.2 ± 16.4 μL vs. 26.7 ± 5.1 μL, *p* = 0.009), showing that although these mice urinated evenly throughout the cage, the larger voids were done in the corner zone.

No significant difference in the inter-marking interval was observed between the groups, initially the frequency of marking was relatively high, after which it gradually decreased (Figure 4C). This could indicate some recurrent scent marking behavior, e.g., re-familiarisation with the environment of the cage (Maruniak et al., 1974).

**Figure 5:**
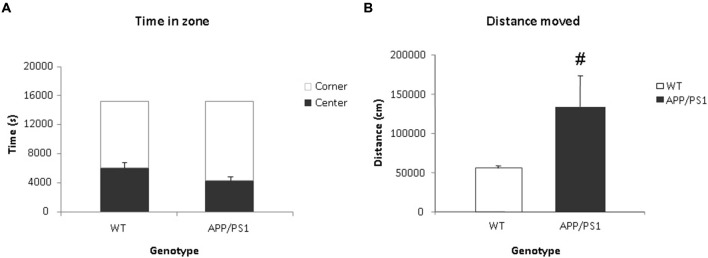
**Voiding behavior task (VBT); Behavioral parameters. (A)** The time spent (in s) in the center and corner zones of the VBT. There was no significant difference in time spent in the center or corner zones between the WT or APP/PS1 groups. **(B)** Mean total distance moved (in cm) in the VBT. The APP/PS1 mice moved significantly more than the WT group. WT *n* = 9 and APP/PS1 *n* = 7. Data represent means ± SEM (^#^*p* < 0.05).

Video tracking indicated that APP/PS1 mice did not spend significantly more time in the center than the WT group (Figure 5A). However, the APP/PS1 mice moved significantly more distance than the WT group (Figure 5B).

### Elevated Zero Maze Task

**Figure 6:**
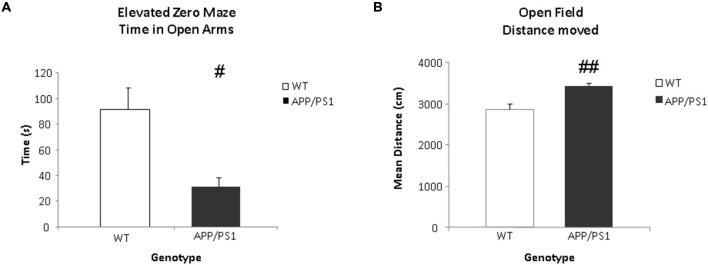
**Elevated zero maze (EZM) and open field (OF) tasks. (A)** The time spent (in s) in the open arms of the EZM. The WT group spends significantly more time in the open arms of the arena (^#^*p* < 0.05). **(B)** The mean total distance moved (in cm) in the OF. The APP/PS1 mice moved more than the WT mice. WT *n* = 9 and APP/PS1 *n* = 7. Data represent means ± SEM (^##^*p* < 0.01).

Anxiety related behavior was examined in the EZM task. This task was conducted one day after the VBT. Data were expressed as time spent in the open arms. Significant differences were found between the groups in the time spent in the open arms. The WT mice spent significantly more time in the open arms of the arena compared to the APP/PS1 mice (*p* = 0.012, Figure 6A).

### Open Field Task

General locomotor behavior was examined using the OF task one day after the EZM task. The data were expressed as total distance moved. The APP/PS1 mice covered significantly more distance compared to the WT mice (*p* = 0.002, Figure 6B).

## Discussion

The association of AD and voiding disorders has been known for a long time (Williams and Pannill, 1982). Currently, no animal data are available linking AD to voiding behavior and/or bladder dysfunction. In this study, we aimed at determining whether the transgenic mice APP/PS1 show alteration of the urinary bladder function and affective behaviour as for the patients suffering from AD. For this, we focused on urinary marking, anxiety-related and general locomotor behavior of a mouse model of AD. To our knowledge this is the first study to describe an altered voiding behavior in a rodent model of AD.

Our results show that the WT mice demonstrated a well-controlled and organized marking pattern with markings occurring almost exclusively in the corner zones of the cage, creating the non-circular marking, a pattern considered to be normal (Gevaert et al., 2007). In contrast, the APP/PS1 mice showed circular markings in the center of the cage. This evenly distributed marking pattern indicates the APP/PS1 mice have no specific preference for location of urination (Figure 3). As urgency urinary incontinence is known to occur in AD (Serra et al., 2010), we hypothesize that these markings could also be a sign of abnormal urination, possibly indicating some form of urinary incontinence.

The results of the EZM show that APP/PS1 mice have elevated anxiety levels, since they spent less time in the open arms of the maze. These APP/PS1 mice are known to have cognitive deficits as well (Vanmierlo et al., 2011), which might contribute to the observed increased anxiety. Importantly, anxiety has been described as one of the most frequent and severe behavioral disturbances in AD and is associated with voiding dysfunction (Fan et al., 2008; Serra et al., 2010). Consequently, increased anxiety levels might therefore explain the altered voiding behavior of the APP/PS1 mice.

In accordance with Vanmierlo et al. (2011), we also found that the APP/PS1 mice moved a significantly larger distance in the arena during both the VBT and the OF tasks. This locomotor behavior in APP/PS1 mice could be similar to the increased wandering behavior as observed in AD patients (Burns et al., 1990). Interestingly, the regions in the brain responsible for locomotor control, e.g., the basal ganglia and the medial frontal lobe, overlap with the frontal micturition center. Lesions in these basal ganglia cause both motor and micturition disorders, a combination which is also seen in AD patients (Garcia-Rill, 1986).

Unexpectedly, in this study, the APP/PS1 mice voided less than WT mice. This could be due to several factors; e.g., increased anxiety suppressing the APP/PS1 mice to void, decreased fluid intake due to behavioral impairment resulted in reduce urine production. In order to minimize the discomfort experienced by animals and the influence of single housing on behavioral outcomes, the mice were housed in group. For this reason, it was not possible to measure the individual water consumption of mice. However, behavioral studies on murine model of Alzheimer’s disease (3 × Tg-AD and APP23 transgenic mice) did not show significant difference in fluid intake between the AD and WT mice (Vloeberghs et al., 2007; Romano et al., 2014).

Another possible explanation is that structural changes in the neural control mechanisms of the lower urinary tract could give rise to aberrant afferent activity leading to an altered voiding behavior. Interestingly, our group has described histological changes in the urinary bladder of the APP/PS1 mice indicating changes at the peripheral level (Biallosterski et al., 2010). In these mice, ganglia were found on an aberrant location, in the bladder wall itself, and the number of intramural afferent nerve fibers was increased (Biallosterski et al., 2010). How and when these changes occurred is still unclear and subjects to further research.

In summary, the current study shows an altered voiding behavior in a mouse model of AD. These alterations in the APP/PS1 mice could be explained by changes in anxiety-related and general locomotor behavior, specific of AD. Indeed previous studies suggest that behavioral changes are an important factor in incontinence in AD (Han and Wang, 2008). However, we hypothesize that the altered voiding behavior is due to multi-factorial changes in behavior as well as in the control of the urinary system leading to “functional incontinence”. To some extend the altered voiding behavior could be caused by an increased level of afferent activity of the lower urinary tract. The mechanisms remain largely unclear but are most likely caused by dysfunctions at both central and peripheral levels of control.

For future research, the non-invasive method of evaluating voiding behavior used in this study should be combined with functional experiments, e.g., using cystometry, and conducted to perform a longitudinal study. This can give more insight into the function and functional changes of the bladder in this animal model and allow to correlate the progression of AD and voiding behavior. Moreover, a newly developed rat model of AD could be an interesting model in terms of translational research to the human condition (Cohen et al., 2013). These models will be useful to elucidate whether functional changes develop first at the peripheral level or at the central level, i.e., due to changes in the bladder itself and/or in the brain in AD. The exact underlying mechanism between AD and altered voiding behavior needs to be elucidated in future research.

## Author Contributions

GK designed the research study. GK and SW contributed essential reagents and tools. BB performed the research. BB and CM analyzed the data. All authors wrote the paper. All authors contributed to and have approved the final manuscript.

## Conflict of Interest Statement

The authors declare that the research was conducted in the absence of any commercial or financial relationships that could be construed as a potential conflict of interest.

## Abbreviations

AD: Alzheimer’s disease
APP/PS1: APP^SL^/PS1^M146L^
EZM: elevated zero maze
OF: open field
VBT: voiding behavior task
WT: wild type.
